# Accuracy of Clinical Diagnosis of Dengue Episodes in the RV144 HIV Vaccine Efficacy Trial in Thailand

**DOI:** 10.1371/journal.pone.0127998

**Published:** 2015-05-26

**Authors:** Punnee Pitisuttithum, Supachai Rerks-Ngarm, Donald Stablein, Peter Dawson, Sorachai Nitayaphan, Jaranit Kaewkungwal, Nelson L. Michael, Jerome H. Kim, Merlin L. Robb, Robert J. O’Connell, In-Kyu Yoon, Stefan Fernandez, Jean-Louis Excler

**Affiliations:** 1 Vaccine Trial Center, Faculty of Tropical Medicine, Mahidol University, Bangkok, Thailand; 2 Department of Diseases Control, Ministry of Public Health, Nonthaburi, Thailand; 3 EMMES Corporation, Rockville, Maryland, United States of America; 4 Royal Thai Army Component, Armed Forces Research Institute of Medical Sciences (AFRIMS), Bangkok, Thailand; 5 Center of Excellence for Biomedical and Public Health Informatics, Faculty of Tropical Medicine, Mahidol University, Bangkok, Thailand; 6 US Military HIV Research Program (MHRP), Bethesda, Maryland, United States of America; 7 The Henry M. Jackson Foundation for the Advancement of Military Medicine, Bethesda, Maryland, United States of America; 8 Department of Retrovirology, US Component—Armed Forces Research Institute of Medical Sciences (AFRIMS), Bangkok, Thailand; 9 Department of Virology, Armed Forces Research Institute of Medical Sciences (AFRIMS), Bangkok, Thailand; International AIDS Vaccine Initiative, UNITED STATES

## Abstract

RV144 was a community-based HIV vaccine efficacy trial conducted in HIV-uninfected adults in Thailand, where dengue virus continues to cause a large number of infections every year. We attempted to document the accuracy of clinically diagnosed dengue episodes reported as serious adverse events (SAEs) and adverse events (AEs) and examine whether dengue serology would support the clinical diagnosis. Subjects without a clinical dengue diagnosis but with an infection or idiopathic fever were selected as a control population. Dengue serology was performed by hemagglutination inhibition on plasma samples. A total of 124 clinical dengue episodes were reported (103 SAEs and 21 AEs). Overall 82.6% of the clinically diagnosed dengue episodes were supported by a positive dengue serology: 71.4% of the AEs and 85.0% of the SAEs. Of the 100 subjects with both clinical dengue and positive serology, all presented with fever, 83% with leucopenia, 54% with thrombocytopenia, and 27% with hemorrhagic symptoms. All episodes resolved spontaneously without sequellae. Only two of 15 subjects with a negative serology presented with fever. The sensitivity and specificity of clinical dengue diagnosis were 90.9% and 74.4%, respectively, when compared to the control population, and with a positive predictive value of 82.6% and negative predictive value of 84.7% when compared to dengue serology. Clinical diagnosis of dengue is an accurate method of dengue diagnosis in adults in Thailand. Large-scale clinical trials offer the opportunity to systematically study infectious diseases such as dengue and other infections that may occur during the trial.

## Introduction

RV144 was a community-based HIV vaccine efficacy trial conducted in HIV-uninfected adults in Rayong and Chonburi provinces, Thailand (2003–2009), where a prime-boost vaccination regimen with ALVAC-HIV (vCP1521) and gp120 AIDSVAX B/E demonstrated vaccine efficacy for prevention of HIV acquisition of 60% after 12 months of follow up, dropping to 31.2% after 42 months (ClinicalTrials.gov NCT00223080) [1, 2]. The vaccine regimen was found to be safe and well tolerated. Of the 16,402 volunteers, 69% of the participants reported adverse events (AEs) and 14.6% experienced serious adverse events (SAEs) any time after the first dose with no significant difference between vaccine and placebo recipients. SAEs coded under ‘Injury and procedural related complications’ were the most common type reported followed by those in the ‘Infection and Infestation’ category [3].

Dengue is the most common mosquito-borne viral disease in the world. In the last 50 years, incidence has increased 30-fold with geographic expansion to new countries and, in the present decade, from urban to rural settings. Dengue poses a substantial economic and disease burden in South East Asia [4].

In Thailand, dengue virus (DENV) continues to cause a large number of infections every year. All four DENV serotypes have co-circulated in the country for many decades. Between 2000 and 2011, Thailand ranked second highest country after Indonesia for dengue-associated morbidity and mortality in South East Asia with peaks in 2001, 2002, 2008 and 2010, disease incidence and death being highest in children <15 years of age, and in Southern provinces [5]. By 2009, the last year of the RV144 study, the dengue morbidity rates per 100,000 inhabitants in the provinces of Chonburi and Rayong were among the highest in the nation. Despite the high rates of DENV infections in these two provinces, the mean age of first time infections increased significantly between 1980 and 2010, presumably due to decreasing birth and death rates among the population [6].

The purpose of our study was to document the accuracy of clinically diagnosed dengue episodes reported as SAEs and AEs which occurred during the course of the community-based HIV vaccine efficacy trial (RV144) as per the 2009 WHO guidelines for dengue diagnosis [7] and, as much as possible, to examine whether dengue serology would support the clinical diagnosis reported by the investigators. To assess this objective, all subjects with an adverse event coded as a DENV infection and available samples were evaluated by dengue serology which was used as gold standard for dengue diagnosis in the current evaluation. To assess the accuracy of clinical dengue diagnosis, an enriched population of subjects with non-dengue SAEs and samples available were selected for dengue serology testing. This enabled estimation of sensitivity, specificity, and the positive (PPV) and negative predictive value (NPV) of dengue clinical diagnosis in the context of a clinical trial that was not focusing specifically on dengue assessments. Selection of the non-dengue subjects is described in the methods section and the criteria utilized were designed to maximize the probability of identifying missed clinical dengue diagnoses.

## Methods

### Population with clinical dengue diagnosis

RV144 participants who were diagnosed with dengue during the course of the trial from October 2003 to June 2009 were retrieved from the trial database used for the safety analysis [3]. Dengue diagnosis was made using the case definition and criteria in the 2009 WHO guidelines [7]. Dengue cases were recorded either as AEs or SAEs. The description of SAEs was recorded on specific SAE record forms while the description of AEs was provided in the clinical file of participants. For the latter, files were retrieved from the clinical trial archives repository in Bangkok. While the description of dengue SAEs was relatively detailed with clinical symptoms and biological assessments, the information on dengue AEs remained limited. Available information collected by investigators was tabulated.

### Control population without clinical dengue diagnosis

All individuals without a dengue diagnosis and an SAE using a preferred term that corresponded to the system organ class “Infections and infestations” or idiopathic fever (pyrexia) occurring between June and September were selected as potential missed dengue cases. A primary list of selected preferred terms included the following: acute tonsillitis, bacteremia, infection, influenza, malaria, meningitis viral, nasopharyngitis, pharyngitis, pyrexia, sepsis, tonsillitis, typhoid fever, upper respiratory tract infection, and viral infection. A secondary list of preferred terms included: scrub typhus, *Plasmodium falciparum* infection, hepatitis A, hepatitis, gastroenteritis, and acute sinusitis. Further criteria included availability of two blood samples (pre and post SAE), exclusion of diagnoses with known cause (e.g., appendicitis, wound, cellulitis, etc.) and individuals whose samples were identified as controls for the immune correlates study [8].

### Plasma samples

Plasma samples of RV144 volunteers were retrieved from the Specimen Processing Laboratory, HIV Center of Excellence, AFRIMS, Bangkok, where they were stored at -80°C. RV144 volunteers had a blood sample collection two weeks post last vaccination and then every six months until the end of the trial. Two samples per volunteer were sorted: a sample prior to the adverse event (either baseline or the closest available prior onset) and the closest available after onset. Samples of volunteers used in the RV144 immune correlates of risk analysis [8] were not used for this study. Plasma aliquots with sufficient volume were sent on dry ice to the Department of Virology, AFRIMS, Bangkok, and stored at -70°C until analysis.

### Dengue serology

Hemagglutination inhibition (HAI) assay was performed by using the method of Clarke and Casals adapted to microplates [9]. Two-fold serially diluted (heat inactivated and acetone extracted) samples were allowed to react with 8 HA units of sucrose-acetone extracted viral antigen. After antibody viral antigen reaction, residual hemagglutination was detected by subsequent addition of goose red blood cells. The highest dilution of sample that inhibited hemagglutination determined the HAI titer of the sample. A 4-fold rise in HAI titer between paired samples to any of the four DENV serotype antigens was defined as DENV infection. HAI assay could not determine the serotype of the infecting virus.

### Statistics

Statistics were primarily descriptive including geometric means and confidence intervals for continuous outcomes and proportions for categorical outcomes. Fisher’s Exact Test was used to compare serology positive rates between AEs and SAEs. Titer ratio was compared between clinical dengue cases and other infectious subjects using the Wilcoxon Rank-Sum Test. Comparison of titer ratio between serology positive clinical dengue and other infectious subjects was performed via the exact Wilcoxon Rank-Sum Test. Sensitivity, specificity, PPV and NPV were calculated using dengue serology as the gold standard and clinical diagnosis of dengue (yes/no) as the diagnostic test of interest. Incidence of dengue was calculated as the number of cases per 100,000 person-years (PY). At risk time was calculated as time from administration of first vaccine through time of last follow-up (calculated as the maximum of last visit (unscheduled, counseling or blood draw) or last reported adverse event).

### Ethics statement

Our study was part of a better characterization of AEs and SAEs in RV144. The RV144 protocol was reviewed by the ethics committees of the Thai Ministry of Public Health, the Royal Thai Army, Mahidol University, and the Human Subjects Research Review Board of the U.S. Army Medical Research and Materiel Command. It was also independently reviewed and endorsed by the World Health Organization and the Joint United Nations Program on HIV/AIDS and by the AIDS Vaccine Research Working Group of the national Institute of Allergy and Infectious Diseases at the National Institutes of Health [1]. For this retrospective study, the amended protocol received approval from the ethics committees of the Thai Ministry of Public Health, the Royal Thai Army, and Mahidol University. Written informed consent was given by all participants that their clinical records and their biological samples could be used for further research after the end of the trial. Volunteers were recorded by their study number only to maintain confidentiality throughout the study.

## Results

### Clinical diagnosis

**Fig 1:**
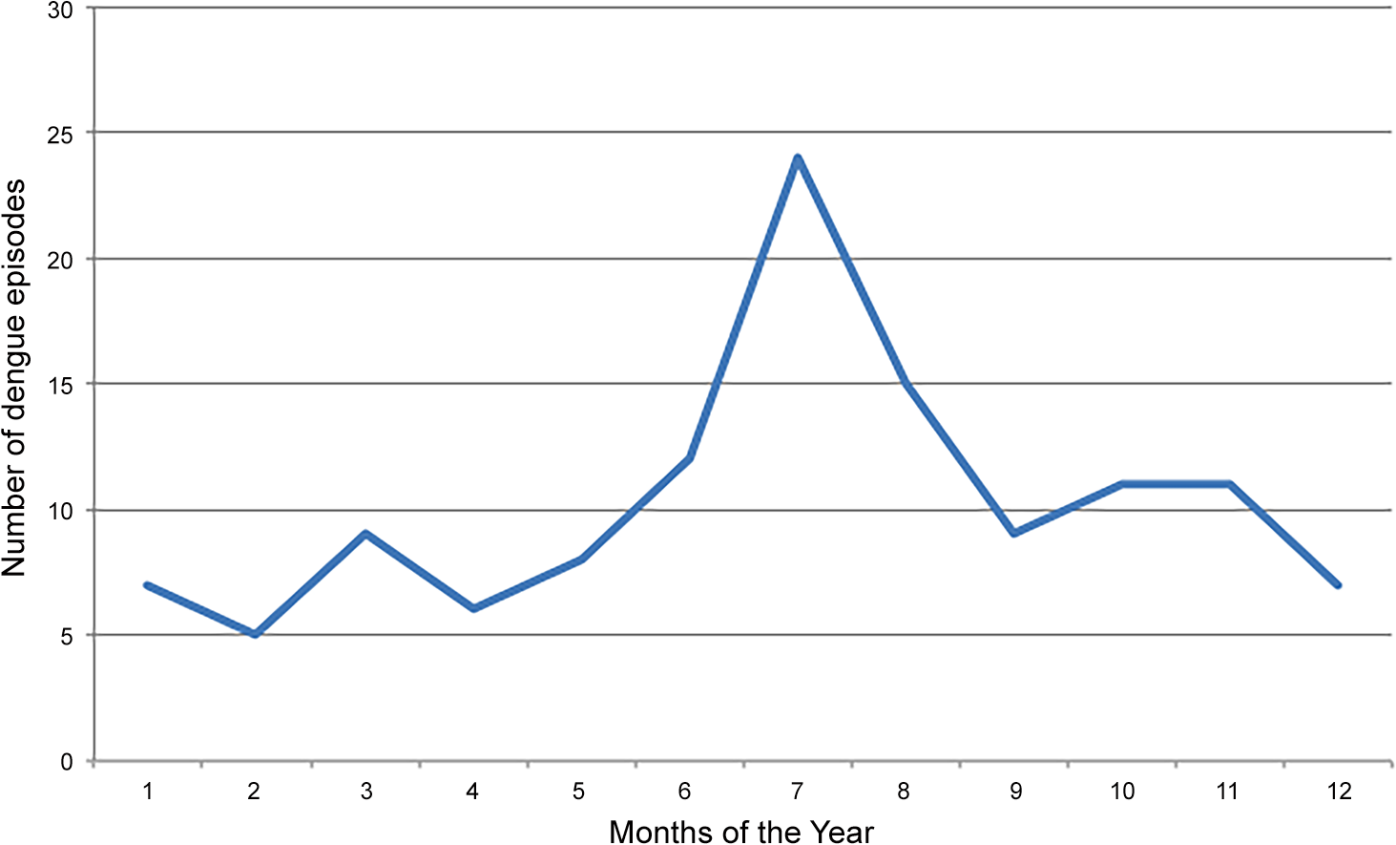
Distribution of the number of clinical dengue episodes over the months of the year during the RV144 trial between July 2004 and November 2008.

**Table 1** provides the demographic characteristics of the RV144 participants with clinically diagnosed dengue episodes and the participants with non-dengue SAEs. A total of 124 clinical dengue episodes (21 AEs and 103 SAEs) were recorded during the duration of RV144. One subject had two clinical dengue episodes (both SAEs) almost 3 years apart resulting in 123 participants with clinical dengue episodes. This represents 4.3% (103/2,394) and 0.19% (21/11,310) of subjects with SAEs and AEs recorded in RV144, respectively [3]. The clinical dengue episodes occurred throughout the year with a peak from June to August (**Fig 1**). The incidence of clinical dengue cases was estimated as 231.9 per 100,000 PY (95%CI 194.5–276.5). The distribution of the AEs and SAEs by severity (as interpreted by the clinician and recorded in the case report form or clinical file) for both clinical dengue episodes and non-dengue SAEs is shown in **Table 2**. Clinical description of AE episodes was not robust since the study required limited documentation for events that were not reported as serious, i.e., requiring hospitalization or life-threatening (symptoms and biological values were available for only 5 of 18 AE subjects with participant binders identified.)

**Table 1:** Demographic Characteristics of RV144 Participants with Clinical Dengue Episodes and Non-Dengue Serious Adverse Events (Controls).

	Clinical Dengue			
	AE	SAE	Controls	Total
Gender				
*Male*	13 (61.9%)	52 (51.0%)	47 (65.3%)	112 (57.4%)
*Female*	8 (38.1%)	50 (49.0%)	25 (34.7%)	83 (42.6%)
Site				
*Phan Thong*	4 (19.0%)	19 (18.6%)	7 (9.7%)	30 (15.4%)
*Si Racha*	2 (9.5%)	16 (15.7%)	14 (19.4%)	32 (16.4%)
*Bang Lamung*	4 (19.0%)	11 (10.8%)	4 (5.6%)	19 (9.7%)
*Sattahip*	0 (0.0%)	15 (14.7%)	7 (9.7%)	22 (11.3%)
*Ban Chang*	4 (19.0%)	10 (9.8%)	12 (16.7%)	26 (13.3%)
*Muang*	2 (9.5%)	11 (10.8%)	9 (12.5%)	22 (11.3%)
*Ban Khai*	3 (14.3%)	8 (7.8%)	9 (12.5%)	20 (10.3%)
*Klaeng*	2 (9.5%)	12 (11.8%)	10 (13.9%)	24 (12.3%)
Treatment Allocation				
*Vaccine*	9 (42.9%)	57 (55.9%)	41 (56.9%)	107 (54.9%)
*Placebo*	12 (57.1%)	45 (44.1%)	31 (43.1%)	88 (45.1%)
Age at Enrollment (yrs)				
*Mean*	23.1	21.7	23.1	22.4
*SD*	3.5	3.2	3.2	3.3
*Median*	23.0	21.0	23.0	22.0

AE: Adverse event; SAE: Serious Adverse Event; SD: Standard Deviation

**Table 2:** Distribution of the Adverse Events (AEs) and Serious Adverse Events (SAEs) for Clinical Dengue Episodes and Other Infectious SAEs During RV144.

	Clinical Dengue Episodes					
	AEs		SAEs		Other Infectious SAEs	
**Severity**	N	%	N	%	N	%
Mild	4	19.0	0	0	0	0
Moderate	12	57.1	6	5.8	2	2.8
Severe	5	23.8	96	93.2	70	97.2
Life-Threatening	0	0	1	1	0	0
**Total**	21	100	103*	100	72	100

*One subject had two episodes of dengue classified as SAEs.

### Dengue serology

Specimens were available for assessment for 121 of the 124 clinical dengue episodes. For the non-dengue SAEs, dengue serology was performed on 72 of the 77 samples identified. The distribution of serology results with geometric mean titer (GMT) ratios between initial and second serology are presented in **Table 3**. Overall 82.6% (100/121) of the clinically diagnosed dengue episodes were serologically positive: 71.4% (15/21) of the AEs and 85.0% (85/100) of the SAEs (p = 0.20). For the non-dengue events, 11/72 (15.3%) were dengue serology positive. Comparing the GMT ratios between dengue and non-dengue events yielded significantly higher GMT ratios in the clinical dengue subjects (p<0.001), which is not unexpected due to the higher serology positive rate. Focusing solely on the serology positive subjects, GMT ratios were again significantly higher in the clinical dengue cases (exact p = 0.037).

**Table 3:** Geometric Mean Titer (GMT) ratio (Post/Pre) and 95% confidence interval (CI) for clinical dengue cases (both SAEs and AEs) and subjects with other infectious Serious Adverse Events (SAEs) displayed by serology dengue status.

Status		N	GMT ratio (Post/Pre)	95% CI
Clinical Dengue (n = 121) *	Serology Negative	21	1.07	0.90–1.27
	Serology positive **	100	48.50	35.03–65.17
Other Infectious SAEs (n = 72)	Serology Negative	61	1.15	1.05–1.25
	Serology positive	11	17.04	5.40–53.78

* Higher GMT ratios in clinical dengue subjects p<0.001; higher GMT ratios in subjects with clinical dengue cases and positive serology

** p = 0.037.

Of the 111 subjects with positive dengue serology (**Table 3**), 100 (90.9%) had an accompanying clinical dengue diagnosis corresponding to a sensitivity of 90.9%. Of the 82 serology negative subjects, 21 (25.6%) received a clinical dengue diagnosis corresponding to a specificity of 74.4%. The predictive values of clinical dengue diagnosis in distinguishing between subjects with true dengue versus those with other infectious complications were 82.6% for PPV and 84.7% for NPV. If the comparison is limited to subjects with a clinical dengue SAE, then the sensitivity was 88.5% (85 clinical dengue of 96 serology positive) and specificity was 80.3% (15 clinical dengue of 76 serology negative) with a corresponding PPV and NPV of 85.0% and 84.7%, respectively.

### Symptoms and serology among clinical dengue episodes

The frequency of clinical symptoms and laboratory parameters evoking dengue as per the 2009 WHO guidelines among the 100 clinical dengue episodes with positive dengue serology is provided in **Table 4**. All subjects presented with fever. Leucopenia (83%) and thrombocytopenia (54%) were other predominant findings observed. Hemorrhagic symptoms were reported in 27% of the subjects, including gingival bleeding, petechiae, epistaxis, and positive tourniquet test (to assess fragility of capillary walls: a blood pressure cuff is applied and inflated to the midpoint between the systolic and diastolic blood pressures for five minutes. The test is positive if ≥10 petechiae per square inch of skin can be counted.) None of the participants experienced shock with neurological symptoms and metabolic disorders. Only two of 21 clinical dengue subjects with negative serology presented with fever: one presented with isolated thrombocytopenia and the other with a positive tourniquet test with unspecified other laboratory values. All episodes resolved spontaneously without sequellae.

**Table 4:** Frequency of symptoms and biological parameters evoking dengue and recorded as clinical dengue episodes with a positive dengue serology.

Symptoms	N = 100
Fever	100
Bleeding signs	27
Thrombocytopenia	54
Leucopenia	83
Elevated liver enzymes	8
Hemoconcentration (elevated hematocrit)	14
Abdominal pain	31

## Discussion

We attempted to characterize SAEs and AEs reported as clinical dengue during a large community-based HIV vaccine efficacy trial and assess the accuracy of the clinical diagnosis in this specific context. The documentation of the frequency of dengue episodes accurately diagnosed clinically contributes to a better knowledge of the background morbidity of the clinical trial population. Clinical data for AE episodes was limited since the study required limited documentation for events that did not meet SAE criteria. Dengue cases represented a minor fraction of the SAEs (4.3%) and AEs (0.19%) reported during the trial. These data are confounded by the fact that RV144 was neither a prospective study of clinical dengue nor a retrospective study using a scoring system [10], and the data herein were collected on case report forms or clinical files by the investigators during the course of the trial. Dengue hemorrhagic fever was diagnosed in 27% of the clinical dengue cases, which corroborates previous findings in patients hospitalized in Bangkok [11]. The clinical symptoms of serology positive, clinical dengue cases provided in **Table 4** match those found in the literature in Thailand [12]. The 2009 WHO severity grading and the frequency of the symptoms could not be precisely determined with the caveats mentioned above. While underreporting of clinical dengue in Thailand is well documented in children [13, 14], Low et al. suggested that in general clinical settings, it is not difficult to identify dengue cases that come to medical attention with high sensitivity but low specificity of clinical diagnosis, in particular at younger age (as volunteers enrolled in RV144) with (98% using WHO 1997 or 2009 sensitivity, but 29% specificity) [15]. While our findings of a high NPV of 84.7% for clinical diagnosis using the 2009 WHO guidelines are in accordance with published values, the high PPV of 82.6% observed sharply contrasts with the low published values when dengue cases are compared to other febrile illnesses (PPV<20%, irrespective of age group) [15]. The reasons of this PPV discrepancy are unclear and may be due to several factors including sample size, control group, context of a clinical trial with clinical staff used to diagnose dengue. The sensitivity and specificity of the 2009 WHO criteria for diagnosing dengue are characterized by their very wide range, depending on several factors that are often unique to the each setting. A recent review found that the 2009 WHO case classification for severe dengue had a sensitivity of 59–98% and specificity 41–99% depending on the study [16]. However, our study differs from these other studies because the clinical dengue cases were identified in the context of a clinical trial and because the dengue serology testing was different from what is classically done for public health surveillance. More specifically, in our study the definition of severity of adverse events differs from that used in the context of public health case classifications. Moreover, the dengue serology is based on seroconversion between 6-month interval samples (primarily assessing HIV-specific immune responses and HIV infection), which contrasts with public health testing for acute illness for which acute and convalescent samples are typically collected. Our findings are mainly applicable to adverse events diagnosed as dengue within the context of a clinical trial.

It remains remarkable that overall 82.6% of the clinically diagnosed dengue episodes were serologically positive. These findings suggest that clinical diagnosis of dengue episode was correctly reported in a majority of cases. Fever cases that were not supported by dengue serology may be attributable to other undiagnosed infectious diseases, in particular Japanese encephalitis (although it is likely that the dengue serology would be positive due to high cross-reactivity among flaviruses in HAI) [17, 18], chikungunya, but also *Salmonella typhi*, rickettsia, and to a lesser extent influenza [19].

The distribution of dengue cases over the year is in agreement with previous findings with a clear peak from June to September. In Thailand, 77% of *Aedes aegypti* may reside indoors and use aquatic larval development sites in or near the home. The rainy season may boost population development by providing needed water in desiccated containers [20]. In our study, the estimated incidence of 231.9 per 100,000 PY is similar to that reported by the Ministry of Public Health of Thailand in 2008 in Chonburi province (141.8–283.5 per 100,000 PY) and lower that in Rayong province (355.3 per 100,000 PY) [21].

## Conclusions

Dengue cases were accurately diagnosed by the RV144 investigators in more than 80% of the reported SAEs and AEs and supported by dengue serology. If dengue serology is considered as the gold standard, the sensitivity and specificity of clinical dengue diagnosis were 90.9% and 74.4%, respectively, with a PPV of 82.6% and a NPV of 84.7%. Large-scale clinical trials offer the opportunity to systematically study infectious diseases such as dengue that may occur during the course of the trial.

### Disclaimer

The opinions herein are those of the authors and should not be construed as official or representing the views of the U.S. Department of Defense or Department of the Army.
